# Nutritional Quality and Health Effects of Low Environmental Impact Diets: The “Seguimiento Universidad de Navarra” (SUN) Cohort

**DOI:** 10.3390/nu12082385

**Published:** 2020-08-09

**Authors:** Ujué Fresán, Winston J. Craig, Miguel A. Martínez-González, Maira Bes-Rastrollo

**Affiliations:** 1 CIBER-Epidemiología y Salud Pública, Instituto de Salud Carlos III, 28029 Madrid, Spain; 2 Instituto de Salud Pública y Laboral de Navarra, 31003 Pamplona, Spain; 3 IdiSNA, Instituto de Investigación Sanitaria de Navarra, 31008 Pamplona, Spain; mamartinez@unav.es (M.A.M.-G.); mbes@unav.es (M.B.-R.); 4 Center for Nutrition, Healthy Lifestyles, and Disease Prevention, School of Public Health, Loma Linda University, Loma Linda, CA 92354, USA; wcraig@llu.edu; 5 Department of Preventive Medicine and Public Health, University of Navarra, 31009 Pamplona, Spain; 6 CIBER-OBN, Instituto de Salud Carlos III, 28220 Madrid, Spain; 7 Department of Nutrition, TH Chan Harvard School of Public Health, Boston, MA 02115, USA

**Keywords:** low impact diet, mortality risk, nutritional quality, dietary environmental impact, land use, water use, energy use, greenhouse gas emission

## Abstract

Current dietary patterns are negatively affecting both the environment and people’s health. Healthy diets are generally more environmentally friendly. However, few studies have focused on the health consequences of diets with low environmental impact. We analyzed differences in the dietary composition (types of food, macro- and micro-nutrients) of those diets with high and low environmental impact, according to greenhouse gas emission and resources use (water, land and energy) using data from a Spanish cohort (17,387 participants), collected by means of a validated food frequency questionnaire. Cox analyses were used to assess the association of dietary environmental impact with total mortality risk. At a given level of energy intake, diets with lower environmental impact contained higher amounts of plant-based foods and lower levels of animal-derived products. Less polluting diets involved higher amounts of polyunsaturated fats and dietary fiber and lower amounts of saturated fats and sodium. However, diets associated with less environmental damage also contained more added sugars, but lower levels of vitamin B12, zinc and calcium. We did not detect any association between dietary environmental impact and risk of mortality. Diets should not only produce minimal environmental impact, but the maximum overall benefits for all key dimensions encompassed in sustainable diets.

## 1. Introduction

Human activities are pushing the environment beyond the limits considered safe for the stability of the earth system and the well-being of humankind [1,2]. Disruption of the environmental conditions may not only lead to altered natural ecosystems, but also entails neglected consequences for humans: damaged infrastructures and human settlements, reduced agricultural yields and freshwater availability, affecting mental health, and increasing human morbidity and mortality [3]. Adaptation strategies are not enough, and additional mitigation measures should be implemented to avoid severe, irreversible consequences [3].

Among all human activities, the current food system is the main driver of both the use of natural resources and the environmental degradation [4,5,6,7,8,9,10,11]. Traditional dietary patterns, mainly plant-based, are being replaced by diets rich in calories, red and processed meat and poor in fruits and vegetables [12,13]. This dietary transition is negatively affecting both the environment and people’s health [14,15,16]. Ultimately, the type and the amount of food people consume directly affects the type and quantity of food produced. In the same way that different food products produce different health effects, they also differ in their impact on the environment [17]. In general, less processed, plant-based products tend to be healthier and more sustainable than animal-based foods [15,17]. Without targeted dietary changes, in addition to health consequences [15], the environmental impact of the food system could reach levels that are beyond the planetary boundaries [18].

By definition, sustainable diets are those diets with low environmental impacts that contribute to food and nutrition security and to healthy life for present and future generations. Sustainable diets are protective and respectful of biodiversity and ecosystems, culturally acceptable, accessible, economically fair and affordable; nutritionally adequate, safe and healthy; while optimizing natural and human resources [19]. Different scientific bodies are placing an increasing impetus upon global actions to achieve human health within the context of sustainable food systems [15,20,21,22]. A growing body of literature is pointing out that the general adoption of sustainable diets, mainly plant-based and low in calories, would lead to a benefit for human and planetary health [23,24,25].

Healthy diets are generally more environmentally friendly [26,27,28]. Indeed, some authors proposed leading with health messages in order to reduce dietary environmental impact, as the promotion of healthy diets would have a dual environmental gain; on the other hand, others have proposed messaging around dietary environmental impact only. However, there is a scarcity of studies focused on assessing the health consequences of diets with low environmental impact. Among those few research studies, the nutritional benefits of low environmental impact diets are unclear. Diets with less environmental damage appear to be low in saturated fat and sodium, and high in fiber. However, they tend to be high in free sugars and low in some essential micronutrients [29,30,31,32]. Fewer studies have focused upon assessing the relationship with healthiness, and that association, if any, is not well established [31]. More research is needed to address this gap in the literature, especially using data of actual food consumption. Therefore, the objective of the current study was to analyze differences in the dietary composition of those diets with high and low environmental impact, as measured by greenhouse gas (GHG) emissions, water use, land use and energy use, according to types of food, macro- and micronutrients, using data from a Spanish cohort. In addition, we assessed the association of diets with high environmental impact with total mortality risk.

## 2. Materials and Methods

### 2.1. Study Population

The Seguimiento Universidad de Navarra (University of Navarra Follow-up) (SUN) project is a dynamic, ongoing and multipurpose Spanish cohort composed of university graduates. Potential participants were informed about the study objective, methodology, data management, and right to drop out of the study without repercussions. Voluntary completion of the baseline questionnaire was considered as giving consent to be a part of the study. The SUN project started in 1999, and follow-up data collection is done every two years by mailed or e-mailed questionnaires. Non-responders to the mailed/emailed follow-up questionnaires were contacted through up to five additional mailings. Further details of the project were published elsewhere [33]. The SUN project is registered in ClinicalTrials.gov with the identification code NCT02669602. The Research Ethics Committee of the University of Navarra approved the protocol on 30 August 2001.

The present analyses were performed by examining the last available database as of the 1 December 2019, corresponding to 22,894 participants. Those with follow-up data of at least 2 years and 9 months were considered in the data analysis; this period allows to recruit exclusively those participants who had spent enough time in the study as to be able to complete and return at least the 2-year follow-up questionnaire (2 years and additional 9 months to account for the lag time in returning the questionnaire). A total of 22,555 participants were recruited up to March 2017 (Supplementary Figure S1). Of these, 5168 were excluded: 2142 had total energy intake outside of predefined limits (<800 Kcal/d or >4000 Kcal/d for men, and <500 Kcal/d and >3500 Kcal/d for women) [34]; 92 women were pregnant at baseline; 1147 reported prevalent diabetes, cardiovascular disease or cancer at baseline; 188 failed to answer 70 or more items on the food frequency questionnaire (FFQ); and 1599 did not answer follow-up questionnaires (retention in the cohort: 91.58%) leaving a total of 17,387 participants available for the data analyses (95.85% of them completed the FFQ in 80–100%).

### 2.2. Dietary Assessment

Habitual diet of the previous year was assessed by means of a semi-quantitative 136-item FFQ, which was completed by participants at baseline. The validity and reproducibility of this questionnaire were previously evaluated [35,36,37]. A typical portion size and 9 consumption frequency categories ranging from “never/almost never” to “more than 6 times/day” were stated for each FFQ item. The product of the consumption frequency by the standard portion size of every item provided the daily food intake. Trained nutritionists computed the nutrient consumption by a computer program based on Spanish food composition tables [38,39]. Missing values were regarded as no consumption.

### 2.3. Assessment of Other Variables

Information about non-dietary variables was also collected at baseline: medical history, sociodemographic factors, lifestyle, and health-related habits. Self-reported data, such as physical activity (total metabolic equivalent of tasks (MET)-hours per week), body mass index (BMI) or hypertension, have been previously validated [40,41,42].

### 2.4. Mortality Assessment

A thorough follow-up ascertainment for deceased participants was done. Participants who did not respond to any of the five follow-up questionnaire mailings were contacted by telephone or email. More than 85% of deaths were reported mainly by relatives, professional associations, and postal authorities. Furthermore, the National Death Index was checked every year to identify deceased cohort members [43].

### 2.5. Environmental Impact Assessment

The environmental impact of participants’ diets was estimated based on a database previously described by Fresán et al. [26]. In short, the GHG emission and the use of resources (water, land and energy) of the production of 1 kg of each food product reported in the FFQ were individually assessed using data previously reported by several institutions and/or research groups (Supplementary Table S1 shows main sources of information). GHG emissions were measured as kg of carbon dioxide equivalents (kg CO_2_e) per kg (kg CO_2_e/kg) of food, water use as liters per kilogram (L/kg) of food, land use as squared meters per kilogram (m^2^/kg) of food and energy use as megajoules per kilogram (MJ/kg) of food. The system boundaries included in the environmental impact assessment were agricultural production and food processing for GHG emission, water use and energy use, and only agricultural production for land use. This database has been previously used in other scientific studies [26,27,28,44].

The environmental impact of each participant’s dietary intake was estimated by summing up, for all the foods in the FFQ, the products of amount consumed per day and the corresponding food’s environmental impact value, for the four indicators considered in the study (i.e., GHG emissions, use of water, land and energy).

### 2.6. Statistical Analysis

Descriptive statistics were used to assess the socio-demographic (sex, age, level of education, marital status), medical and lifestyle (BMI, hypertension, hypercholesterolemia, smoking status, physical and sedentary activities) characteristics of the cohort. Data about dietary environmental impact (GHG emissions, water use, energy use and energy use) of the cohort was also presented. Participants were classified into quartiles according to GHG emission of their diet, and same information was described according to these quartiles.

The composition of participants´ diet was assessed by means of the consumption of several food groups, and the intake of selected macro and micronutrients. Supplementary Table S2 shows specific food products, and their respective serving sizes, considered in each food group. Differences in energy intake could lead to a higher/lower nutrient intake due to the over- or under-consumption of food, and not due to the quality of the diet [45]. In addition, excessive consumption could lead to a higher environmental impact [46]. Our main interest was assessing the relationship between the environmental impact and the quality of the diet. To rule out the influence of energy consumption, the four environmental indicators were adjusted for energy intake, for men and women separately, using the residual method [45]. Therefore, when evaluating the composition of the diet in light of its environmental impact, participants´ diet were compared according to energy-adjusted quartiles of the environmental indicators. Linear regression was used to assess differences in GHG emissions, use of resources and dietary components between all four quartile groups, with the first quartile as the reference group. Linear trends between quartiles were tested. Analysis of variance (ANOVA) tests were utilized to compare quartiles.

Cox regression models were utilized to determine the relationship between quartiles of dietary environmental impact and all-cause mortality during follow-up. Hazard ratios (HR) and their 95% confidence interval (95% CI) were calculated using the lowest quartile as the reference category. Age was the underlying time variable. Date of death was used as the outcome time. Participants who did not complete follow-up questionnaires and whose deaths have not been documented were censored when last follow-up questionnaire was completed, using the date as exit time. We fitted an age- and sex-adjusted model, stratified by 10 categories of age and 4 categories of year of entrance to the cohort. We also fitted a multivariable model additionally adjusted for the following potential confounders: BMI, a quadratic term for BMI, smoking status, physical activity, hours spent watching TV, prevalent hypertension, prevalent hypercholesterolemia and marital status. Linear trends were analyzed by introducing the median of the quartile as a continuous variable in the models and calculating the p-value. In addition, multivariable-adjusted estimates for restricted cubic splines were used to evaluate the association between dietary environmental impact and mortality risk. Sensitivity analyses were conducted by refitting the model under different assumptions to assess the robustness of the results: excluding participants with energy intake under percentile 1 or over percentile 99; excluding participants who died in the first 4 years of follow-up (because a short follow-up period could not be sufficient to observe a relationship between the diet composition and health outcomes); censoring follow-up at 10 years (because late deaths could not be related to baseline diet); excluding deaths occurring at ≤ 45 years (because mortality risk could not be accurately assessed in young persons); and excluding deaths occurring at > 80 years (because mortality risk is certainly influenced by aging). Moreover, stratified analysis according to age, sex, baseline BMI and physical activity were performed. Interactions were assessed through a likelihood ratio test.

Main analyses were performed using GHG emissions, but water use, land use and energy use were also considered as environmental impact indicators in sensitivity analyses. Correlation among quartiles of the four environmental impact indicators was assessed by Pearson coefficients.

All p-values presented are two-tailed; *p* < 0.05 was considered statistically significant. Analyses were performed using STATA/SE V.12.1 [47].

## 3. Results

### 3.1. Baseline Characteristics of the Cohort

According to Table 1, participants of the current study were relatively young (mean age: 37 years old), mainly women (61%) and highly educated persons. On average, they were generally healthy, active, with a BMI suggesting normal weight (23.5 kg/m^2^), and an average energy intake of around 2300 kcal per day. Among them, there was a low proportion of current smokers (22%) and low prevalence of hypertension or hypercholesterolemia. On average, the daily dietary GHG emissions and use of resources (water, land and energy) of the cohort were 3.54 (min-max: 0.32–14.62) kgCO_2_e, 3753 (407–12,160) L, 7.17 (1.31–23.38) m^2^ and 17.59 (2.52–48.47) MJ, respectively.

Participants classified in the lowest quartile of the dietary GHG emissions were more likely to be women, older and married participants. The prevalence of people with hypertension and hypercholesterolemia was higher in the lowest quartile, while less people were current smokers. They tended to have lower caloric intake and being more active. The diet of the participants with lower GHG emissions were also less resource consumptive.

### 3.2. Food Consumption and Nutrient Intake

**Figure 1:**
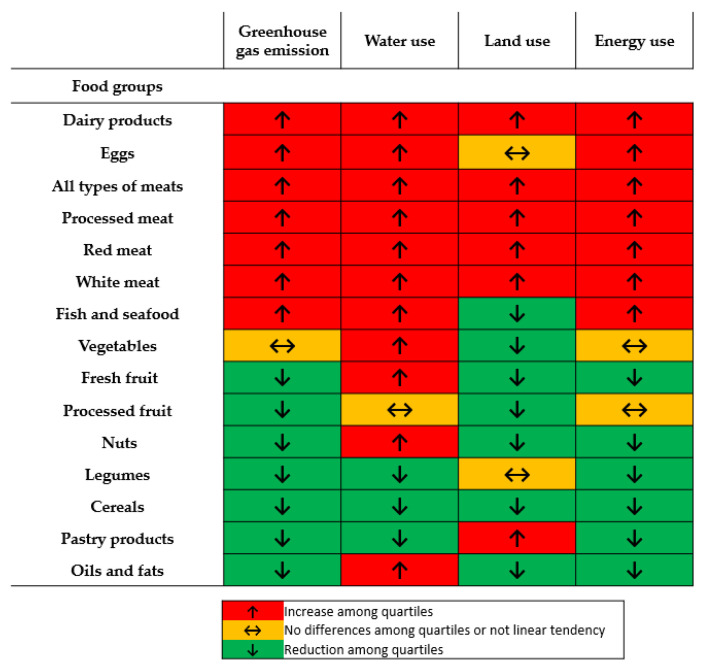
Tendencies in food groups’ consumption among energy-adjusted quartiles of the indicated environmental impact indicators. Specific food products, and their serving sizes, collected in each food group is described in Supplementary Table S2.

After adjusting for energy, diets with lower GHG emission were significantly lower in animal-derived products, such as dairy, eggs and any type of flesh product (Table 2). At the same time, those diets were higher in plant-based products, such as fruits, nuts, legumes and cereals (including pastry products), and also in oils and fats. When considering the dietary environmental impact based on land use and energy use, besides little discrepancies, the same general pattern was observed: diets with lower use of resources contained lower amounts of animal products and higher of plant-based foods. However, in relation to water use, more remarkable tradeoffs were detected. Plant-based products, like vegetables, fruits, nuts and oils, were consumed in diets with higher use of water (Figure 1 and Supplementary Tables S3–S5).

**Figure 2:**
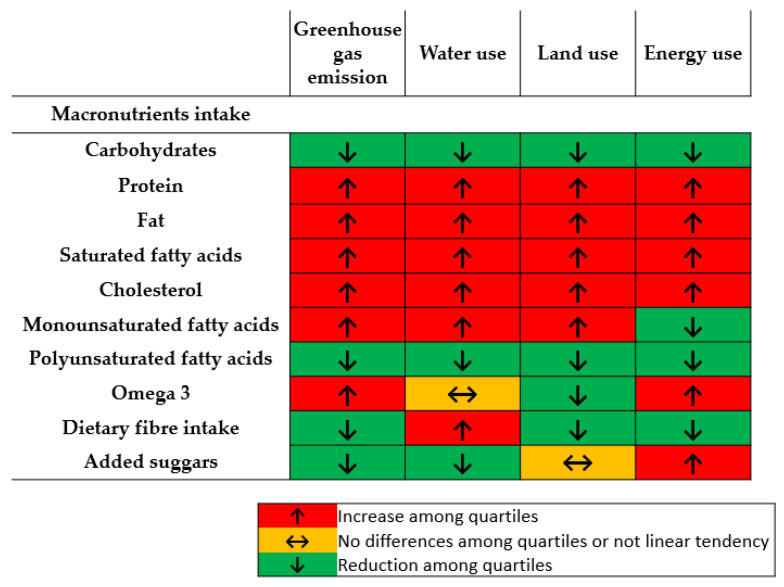
Tendencies in macronutrients intake among energy-adjusted quartiles of the indicated environmental impact indicators.

Higher intake of carbohydrates, polyunsaturated fatty acids, although not omega-3, dietary fiber and added sugars was observed among the diets with lower GHG emissions (Table 2). On the other hand, those diets were associated with a lower intake of protein and fats, both saturated and monounsaturated fatty acids. Agreements among indicators confirmed that diets with lower environmental impact would contain higher quantities of carbohydrates and polyunsaturated fatty acids, while lower amount of proteins and fats, specially saturated fatty acids. If not considering the water use indicator, diets with lower environmental impact would also contain higher amount of fiber (Figure 2 and Supplementary Tables S3–S5).

**Figure 3:**
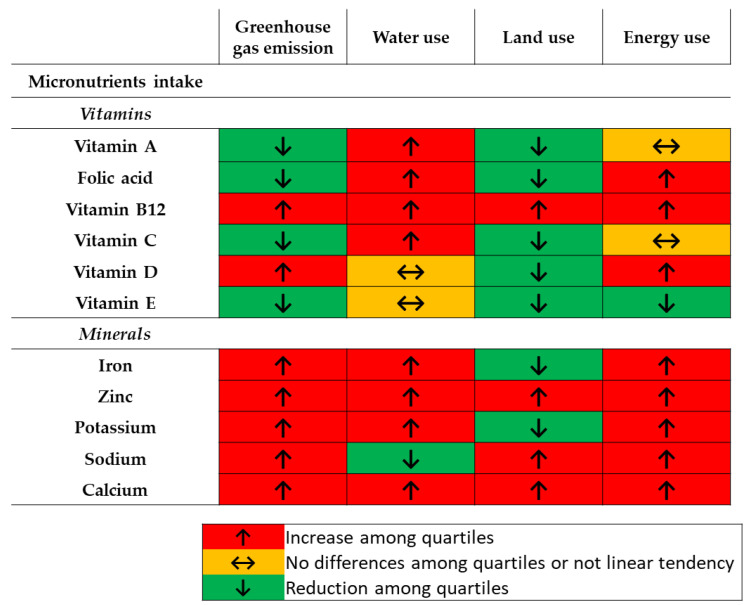
Tendencies in micronutrients intake among energy-adjusted quartiles of the indicated environmental impact indicators.

Diets with lower GHG emissions contained lower amounts of some vitamins (B12 and D) and all analyzed minerals (iron, zinc, potassium, sodium and calcium), and higher levels of vitamins A, C, E and folic acid (Table 2). Several inconsistencies appeared in relation to micronutrient intake when considering several indicators. Overall, less polluting diets would contain lower amount of vitamin B12, and minerals such as zinc and calcium. When leaving out the indicator water use, those diets would also contain higher amount of vitamin E, while lower of sodium (Figure 3 and Supplementary Tables S3–S5).

### 3.3. Dietary Environmental Impact and Mortality Risk

A total of 305 deaths were identified during the follow-up period (202,573 person-years), with a median follow-up of 12.25 years. No significant association between dietary GHG emission and mortality risk was observed whether comparing quartiles or using restricted cubic splines (Table 3 and Supplementary Figure S2). The main results did not substantially change when considering other environmental indicators (Table 3) or in any of the different scenarios checked in the sensitivity analyses (Table 4), confirming their robustness. In the subgroups analyses, the results obtained were in the same direction as the main analysis (Supplementary Table S6). No evidence of interaction was detected for age, sex, BMI nor physical activity (*p* = 0.099, 0.849, 0.925 and 0.472, respectively).

### 3.4. Correlation Among Environmental Impact Indicators

A general agreement among indicators was observed, and those diets with higher environmental impact according to one indicator were also more polluting and resource consumptive for the other indicators (Table 2 and Supplementary Tables S3–S5). Pearson correlation analysis showed high correlation among quartiles of the environmental impact indicators. Coefficients ranged from 0.70 to 0.81 (Supplementary Table S7).

## 4. Discussion

On the basis of food-consumption data from a Spanish cohort, the current study showed that, at a given level of energy intake, diets with lower environmental impact contained higher amounts of plant-based foods and lower amounts of animal-derived products. In addition, less environmentally polluting diets could be considered as having a good nutritional profile because they contained higher quantities of well-known nutrients, such as polyunsaturated fatty acids and dietary fiber, and lower unhealthy nutrients, like saturated fatty acids and sodium. However, at the same time, the intake of added sugars was higher, while of some vitamins and minerals, such as vitamin B12, zinc and calcium, were lower. We did not detect any association between dietary environmental impact and risk of mortality. Besides a general agreement considering different environmental impact indicators, trade-offs could be detected, emphasizing the necessity of considering dietary environmental impact based on different footprints in order to accomplish a more comprehensive assessment.

A general agreement among environmental indicators has been previously described, suggesting that foods that have a low environmental impact for one indicator often have a low impact for others [17]. It is well recognized that, in general, plant-based products are less environmentally damaging and resource consumptive than animal-derived foods [17,48,49]. Although small amounts of animal products could be consumed within the framework of an overall low impact diet [26,28], plant-based diets are generally less polluting on the environment [24,27,49,50,51]. In fact, moving away from current dietary patterns high in animal-based products to diets rich in plant-based products would have the potential to notably decrease the environmental impact of the food system [18,23,25]. Therefore, it is not surprising that, in our study, those diets with a lower environmental impact for a given energy intake were rich in plant-based products and low in animal-derived ones. However, our results suggest that the concordance among indicators is not always the case. For example, we observed that consuming some plant-based food groups, namely vegetables, fruits and nuts (but not cereals and legumes), was associated with increased water usage. This observation seems reasonable considering the different farming methods used. While vegetables, fruits and nuts are commonly irrigated crops, legumes and cereals are rain-fed crops. Therefore, the former utilizes a higher water usage from watersheds. Not only the type of product but also different agricultural techniques used could result in different dietary environmental impacts [52].

The observed intake of macro- and micro-nutrients was in accordance with the food groups commonly consumed in diets with lower environmental impact. It should be noticed, however, that with the exception of fiber and vitamin B12 which can only be naturally obtained from plant-based products and animal-sourced products, respectively, all macronutrients, and vitamins and minerals can be found in both plant and animal products. Nevertheless, specific nutrients are more frequently obtained from one or the other. Addressing the macronutrient composition, the fact that low environmental impact diets were generally rich in carbohydrates, polyunsaturated fatty acids, dietary fiber and added sugars, and lower in protein and monounsaturated and saturated fatty acids, is to be expected for predominantly plant-based diets. Firstly, plant-based products are the only dietary source of fiber. Using water use as the indicator, the intake of dietary fiber was lower, in accordance with a lower consumption of some plant-based food groups in diets with lower water use. Secondly, we observed that diets with low GHG emissions and water use contained higher amounts of added sugars. Sugar-producing crops are among the less polluting produce [49]. However, a different picture emerges when energy usage is considered. Added sugars mainly come from processed foods. Our results are explained by the fact that the energy usage correlates with the level of food processing. While legumes, nuts and cereals have a good protein content, other plant-based products, such as vegetables and fruits, provide less protein. In general, the protein level tends to be higher in animal-based foods [53]. Animal protein is typically associated with substantial levels of fat, especially saturated fat, and processed meat products are high in sodium [53], in accordance with our findings. It should be noted that participants consuming diets with a higher environmental impact consumed lower amounts of added fats and oils. However, their diets contained higher levels of fat, indicating that their main dietary sources of fat were animal products, and not externally added fat.

Regarding micronutrient intake, a lower intake of some vitamins and minerals was associated with less polluting and resource-consuming diets, especially vitamin B12, zinc, sodium and calcium. Aside from fortified products, vitamin B12 is exclusively found in animal food sources, especially red meat. Animal proteins tend to be richer sources of zinc, while dairy is a good source of calcium [54]. Therefore, it is reasonable to assume that diets lower in both environmental impact and animal products, contain lower levels of these vitamins and minerals. If leaving aside water use indicator, there was general agreement about a higher intake of vitamins A, C and E. Although animal products have considerable levels of vitamin A, it is also widely available among plant foods, in the form of various carotenoids (a precursor of vitamin A) [54]. In Spain, fruits and vegetables are the main source of vitamin C, while vegetable oils, fruits, nuts and seeds are good sources of vitamin E [55]. Indeed, we observed that the intake of these two vitamins, C and E, was lower in diets with lower water use, suggesting their plant origin.

Altogether, our findings indicate that diets with lower environmental impact may not necessarily show an overall a priori better nutritional profile in every aspect: healthy features were seen in less environmentally damaging diets, such as plant-based foods, having a higher intake of dietary fiber and some vitamins, and lower level of fats, especially saturated fatty acids, and sodium. Nevertheless, at the same time, these diets were seen higher in added sugars, and lower in vitamin B12, zinc, and calcium. Although statistically significant differences in the consumption of food groups, and macro and micronutrient intake between diets with different environmental impact were observed in the current study, it does not imply that the differences would be clinically relevant. Values reported here were about quantities of macro and micronutrients in the diet. The bioavailability of some nutrients, such as iron and zinc can vary depending upon whether they come from animal- or plant-based foods [56,57]. In addition, interactions may exist among nutrients, influencing their absorption, in addition to the effect of culinary techniques. For instance, vitamin C facilitates iron absorption, while iron absorption can be hindered in the presence of phytates and calcium [58,59,60]. It is worth also highlighting the role of the own physiological characteristics of each human being in nutrient assimilation. Additionally, it should be noticed that higher intake of some micronutrients does not necessarily indicate better nutritional profile of the diet or health benefits; for example, a sustained high dietary calcium intake could suppose a cardiovascular risk [61]. Altogether, an increasing emphasis is being focused on assessing the adequacy of the whole diet according to health effects, rather than focusing upon food groups or nutrient profile [62]. Indeed, when assessing the health effects of the diets based on their environmental impact no association with mortality was observed. Therefore, diets with lower environmental impact did not necessarily show effects on longevity. Our results could be considered robust because the findings were confirmed in a variety of sensitivity analyses and in view of four specific environmental impact indicators. Besides participants with lower dietary environmental impact consumed greater amounts of healthy plant-based foods, their intake of added sugars was also higher. A high consumption of ultra-processed foods, rich in added sugars, has been reported to be associated with higher mortality risk in our cohort [63]. Thus, a higher consumption of ultra-processed products could partly be responsible for the lack of association between dietary environmental impact and risk of mortality.

Our findings are in accordance with previous investigations assessing dietary environmental impact. However, most of the studies only considered GHG emissions, and just a few focused on others indicators [24,25]. Therefore, further studies are needed, such as the current one, presenting dietary environmental impact considering different footprints. A recent review suggested that diets with lower GHG emissions tended to be lower in animal products, saturated fat and salt [31]. Yet these diets were often high in added sugars. Dietary scenarios that have lower environmental impact compared with average consumption patterns may not result in improvements in overall nutritional quality. Payne et al. [31] also reported that less polluting diets were generally lower in a variety of micronutrients. A general agreement among studies about the lower intake of zinc, calcium and vitamin B12 in those diets was described. The studies considered in that review were a mix of investigations, based not only on real consumption, but also on theoretical data, including investigations addressing dietary replacements. Focusing on research based on real food consumption, the same general agreement emerged about some attributes of diets with lower GHG emissions: they are mainly plant-based, with a low amount of animal products, especially red meat; they contain a higher amount of dietary fiber and added sugars, and less saturated fatty acids and proteins; and they are lower in a number of vitamins and minerals, especially vitamin B12, calcium, zinc and sodium [30,32]. Taking an overall view of the diet, Vieux et al. [32] observed that those diets with the lowest diet-related GHG emissions did not have the highest nutritional quality. Meanwhile, Rose et al. [30] observed that diets with lower GHG emissions did not necessarily score higher in all components of the healthy eating index. The WHO identified the necessity of limiting sugar, salt and saturated fat in order to reduce non-communicable diseases [64]. Micronutrient deficiencies are also involved with health problems worldwide [65]. Therefore, dietary recommendations for reducing dietary environmental impact must also address sugar and saturated fat consumption while assuring appropriate micronutrient intake.

Few studies have been aimed at assessing the health outcomes of less environmentally polluting diets [25,31]. Among them, inconsistent findings were reported, and it has been stated that the magnitude of health effects does not show a statistical association with that of environmental benefit [25]. While some reports have associated diets with lower GHG emissions with lower risk of cancer and mortality, others have found that following those diets would increase cancer, cardiovascular and mortality risk [31]. However, the number of studies is small, and it cannot be ruled out that the lack of studies reporting no association, neither positive nor negative, as we are doing here, is due to publication bias.

The definition of sustainable diets stresses the necessity of considering several aspects when promoting dietary patterns –the environment, nutrient adequacy, culture, accessibility or economic affordability- without prioritizing one over the others. Although some trade-offs among these aspects exist [27], recent publications show that diets that consider several dimensions, such as nutrient adequacy, environmental impact, and affordability, could be culturally acceptable and accessible and still be promoting health benefits [44,66,67]. Forthcoming dietary studies should take a comprehensive point of view if a greater knowledge of sustainable diets and their implementation is the objective [68].

Some limitations of the current study could be mentioned. The system boundaries considered in the environmental impact database only included food production and processing, leaving out other phases of the food system, such as packaging and transportation. However, food production is by far the most polluting phase of the food system [69]. In addition, the environmental impact of foods was determined based exclusively on conventional production practice data, and within each indicator, contemplating different practices. In addition, not all data collected was Spanish-specific. Fortification was not considered when establishing the nutrient profile of foods. Its consideration would lead to different results. Nevertheless, this study goes beyond nutrients and assesses health effects. In any case, no association was observed between dietary environmental impact and mortality. Food consumption was self-reported making it susceptible to information bias. Our cohort is composed of healthy, young university graduates. This cohort could be seen as thoughtful consumers, with above-average knowledge about healthy and sustainable eating habits. Certain eating habits, typical of representatives of other social groups, were not necessarily included. Thus, our cohort could not be considered as representative of the population of the whole country. However, similar findings of nutrient profiles were observed in other cohort studies, both European and from the United States [30,32]. Some strengths of the current study are the use of a well-known and relatively large cohort with high long-term retention rate and the previously repeated validation of the semi-quantitative FFQ, which assesses the consumption of more than 130 foods over a wide frequency range. The characteristics of our cohort, high educational level and commitment of participants, ensures internal validity, by means of high quality and improved accuracy in self-reported data, a better classification of health and disease conditions, and a reduction in the potential for confounding due to socio-cultural factors, given the homogeneity of the cohort. Though some food products recently introduced into the market are not considered in the FFQ, such as fortified plant-based milks or veggie meats, respondents had the opportunity to mention any food commonly consumed and not mentioned in the FFQ in open-answered questions. Additionally, the analyses were controlled for a good number of potentially confounding variables and sensitivity analyses were performed, confirming the robustness of the results. Most of the previous research having the same objective was mainly focused on assessing the nutrient profile, and exclusively considering dietary environmental impact by GHG emission. We expanded our investigation beyond nutrient intake, to assess health outcomes (mortality), and the environmental impact to other dimensions beyond GHG emissions.

## 5. Conclusions

The current study shows that diets with lower environmental impact may not necessarily show an overall better nutritional profile in every aspect: a priori healthy features (preference for plant-based products, higher intake of dietary fiber, and lower intake of saturated fatty acids and sodium) coexist with higher levels of added sugars, and lower levels of B12, zinc and calcium. Beyond nutritional profile, no association between dietary environmental impact and mortality risk was observed. Sustainable diets are being encouraged for the sake of people, environment and societies. Our findings emphasize the necessity of designing diets with respect to not only the minimum environmental impact, but also considering the maximum overall benefit for all key dimensions encompassed in sustainable diets, and even to extend it to sustainable food systems.

## Figures and Tables

**Table 1:** Main sociodemographic, medical and lifestyle characteristics of the 17,387 participants of the Seguimiento Universidad de Navarra (SUN) cohort assessed in the current study, 1999–2019.

	Overall Cohort	Greenhouse Gas (GHG) Emission			
		Q1	Q2	Q3	Q4
Frequency (n)	17,387	4347	4347	4347	4346
**Sociodemographic data**					
Women (%)	61	65	63	62	54
Age, years, mean (SD)	37 (12)	38.8 (12)	37.5 (11.9)	36.7 (11.5)	35.7 (11.4)
Educational level (%)					
Technical	5	4	5	6	7
Graduated	76	76	76	76	75
Master/doctoral	19	20	18	18	19
Civil status (%)					
Single	45	43	44	45	49
Married	50	50	51	50	47
Others	5	7	5	5	4
**Medical and lifestyle data**					
Body mass index (kg/m^2^), mean (SD)	23.5 (3.5)	23.3 (3.4)	23.4 (3.4)	23.4 (3.5)	23.7 (3.5)
Hypertension (%)	6	7	7	6	6
Hypercholesterolemia (%)	16	19	17	15	14
Smoking status (%)					
Current smoker	22	20	21	22	26
Former smoker	28	30	30	28	26
Daily energy intake (Kcal/d)	2356 (610)	1821 (466)	2219 (460)	2501 (470)	2883 (486)
Physical activity (METs-h/day)	3.10 (3.30)	2.98 (3.08)	2.98 (3.00)	3.09 (3.26)	3.41 (3.71)
Hours watching TV (h/day)	1.60 (1.20)	1.56 (1.15)	1.59 (1.13)	1.6 (1.2)	1.63 (1.19)
**Dietary environmental impact data**					
GHG emission (kg CO_2_e/day), mean (min-max)	3.54 (0.32–14.62)	2.25 (0.32–2.78)	3.13 (2.79–3.45)	3.81 (3.46–4.18)	4.97 (4.19–14.62)
Water use (L/day), mean (SD, min-max)	3753 (407–12160)	2680 (407–5613)	3447 (1192–7461)	4022 (2060–6882)	4866 (2752–12160)
Land use (m^2^/day), mean (SD, min-max)	7.17 (1.31–23.38)	5.29 (1.31–21.12)	6.68 (2.28–23.38)	7.65 (3.54–18.65)	9.05 (3.58–16.62)
Energy use (Megajoules/day), mean (SD, min-max)	17.59 (2.52–48.47)	12.91 (2.52–29.41)	16.25 (8.21–33.10)	18.66 (9.62–36.55)	22.55 (12.71–48.47)

METs: Metabolic equivalent of task. CO_2_e: carbon dioxide equivalents.

**Table 2:** Environmental impact, food group consumption and nutrient intake of the 17,387 participants of the SUN cohort assessed in the current study, 1999–2019, according to energy-adjusted quartiles of the dietary greenhouse gas (GHG) emissions.

	Energy-Adjusted GHG Emission (kg CO_2_e/d)					
	Q1	Q2	Q3	Q4	*p* Trend *	*p* Value ⱡ
	0.05–3.00	3.01–3.48	3.49–4.01	4.02–13.21		
Frequency (n)	4347	4347	4347	4346		
**Environmental impact data**						
GHG emission (kg CO_2_e /day), mean (SD)	2.67 (.73)	3.16 (.74)	3.69 (.71)	4.64 (1.03)	<0.001	<0.001
Water use (L/day), mean (SD)	3348 (908)	3485 (920)	3815 (864)	4368 (953)	<0.001	<0.001
Land use (m^2^/day), mean (SD)	6.79 (2.01)	6.79 (1.91)	7.21 (1.80)	7.89 (1.94)	<0.001	<0.001
Energy use (Megajoules/day), mean (SD)	15.7 (4.3)	16.6 (4.1)	17.8 (4)	20.3 (4.8)	<0.001	<0.001
**Food (servings/day) ^a^**						
Dairy products	2.66 (1.51)	2.9 (1.48)	2.98 (1.57)	3.43 (2.02)	<0.001	<0.001
Eggs	2.64 (0.99)	2.74 (0.87)	2.78 (0.87)	2.81 (0.90)	<0.001	<0.001
All types of meats	1.30 (0.68)	1.68 (0.70)	1.98 (0.72)	2.46 (0.92)	<0.001	<0.001
Processed meat	0.28 (0.18)	0.42 (0.21)	0.58 (0.23)	0.80 (0.33)	<0.001	<0.001
Red meat	0.23 (0.18)	0.29 (0.19)	0.32 (0.22)	0.39 (0.30)	<0.001	<0.001
White meat	0.79 (0.55)	0.96 (0.59)	1.07 (0.63)	1.27 (0.76)	<0.001	<0.001
Fish and seafood	0.65 (0.41)	0.69 (0.41)	0.72 (0.41)	0.80 (0.48)	<0.001	<0.001
Vegetables	2.74 (1.59)	2.60 (1.39)	2.59 (1.45)	2.74 (1.62)	0.802	<0.001
Fresh fruit	2.81 (2.37)	2.37 (1.88)	2.24 (1.77)	2.07 (1.61)	<0.001	<0.001
Processed fruit	0.14 (0.28)	0.10 (0.22)	0.09 (0.18)	0.09 (0.23)	<0.001	<0.001
Nuts	0.22 (0.33)	0.14 (0.21)	0.12 (0.18)	0.11 (0.17)	<0.001	<0.001
Legumes	0.40 (0.35)	0.37 (0.28)	0.37 (0.26)	0.37 (0.27)	<0.001	<0.001
Cereals	2.38 (1.55)	1.92 (1.23)	1.77 (1.10)	1.49 (1.02)	<0.001	<0.001
Pastry products	1.29 (1.19)	0.99 (0.84)	0.93 (0.76)	0.82 (0.68)	<0.001	<0.001
Oils and fats	2.31 (1.77)	1.93 (1.54)	1.77 (1.41)	1.59 (1.31)	<0.001	<0.001
**Macronutrient intake**						
Carbohydrates (% of energy)	48 (7)	44 (6)	42 (6)	39 (7)	<0.001	<0.001
Protein (% of energy)	16 (2)	18 (3)	19 (3)	20 (3)	<0.001	<0.001
Fat (% of energy)	35 (7)	36 (6)	37 (6)	39 (6)	<0.001	<0.001
Saturated fatty acids (% of energy)	11 (3)	12 (3)	13 (3)	14 (3)	<0.001	<0.001
Cholesterol (mg/d)	354 (127)	390 (125)	428 (137)	491 (165)	<0.001	<0.001
Monounsaturated fatty acids (% of energy)	15 (4)	15 (4)	16 (3)	17 (3)	<0.001	<0.001
Polyunsaturated fatty acids (% of energy)	5.3 (1.8)	5.2 (1.6)	5.1 (1.4)	5.1 (1.4)	<0.001	<0.001
Omega 3 (mg/d)	2.61 (1.35)	2.52 (1.18)	2.60 (1.17)	2.77 (1.19)	<0.001	<0.001
Dietary fiber intake (g/day)	32.2 (13.9)	27.3 (11.1)	26.3 (10.9)	25.7 (11.2)	<0.001	<0.001
Added sugars (g/day)	57.8 (31.3)	54.2 (28)	54.3 (28)	55.9 (32.4)	0.013	<0.001
**Micronutrient intake**						
*Vitamins*						
Vitamin A (μg/d)	2121 (1599)	1916 (1304)	1887 (1311)	1957 (1482)	<0.001	<0.001
Folic acid (μg/d)	427 (184)	399 (166)	398 (165)	412 (176)	<0.001	<0.001
Vitamin B12 (μg/d)	7.85 (4.05)	8.90 (4.26)	9.78 (4.53)	11.41 (5.87)	<0.001	<0.001
Vitamin C (mg/d)	301 (169)	278 (150)	270 (140)	271 (149)	<0.001	<0.001
Vitamin D (μg/d)	5.97 (4.32)	5.95 (4.10)	6.08 (4.20)	6.58 (4.93)	<0.001	<0.001
Vitamin E (mg/d)	7.92 (4.41)	6.76 (3.41)	6.56 (3.01)	6.57 (2.95)	<0.001	<0.001
*Minerals*						
Iron (mg/d)	17.3 (5.3)	16.4 (4.9)	16.7 (4.8)	17.7 (5)	<0.001	<0.001
Zinc (mg/d)	16.4 (10.2)	17.1 (10.4)	17.8 (10.4)	20.0 (12.1)	<0.001	<0.001
Potassium (mg/d)	4841 (1694)	4626 (1497)	4686 (1493)	4934 (1545)	<0.001	<0.001
Sodium (mg/d)	3143 (2072)	3193 (1978)	3326 (2110)	3640 (2587)	<0.001	<0.001
Calcium (mg/d)	1168 (423)	1186 (411)	1207 (436)	1337 (560)	<0.001	<0.001

* Linear trends were analyzed by introducing the quartiles as continuous variables. *p* < 0.05 was considered statistically significant. ⱡ Analysis of variance (ANOVA) tests were utilized to compare quartiles. *p* < 0.05 was considered statistically significant. ^a^ Specific food products, and their serving sizes, collected in each food group is described in Supplementary Table S2.

**Table 3:** Risk for all-cause mortality (hazard ratio and 95% confidence intervals) of the 17,387 participants of the SUN cohort assessed in the current study, 1999–2019, according to energy-adjusted quartiles of the indicated dietary environmental impacts.

	**Greenhouse Gas Emission**				*p* for trend
	**Q1**	**Q2**	**Q3**	**Q4**	
N	4347	4347	4347	4346	
Deaths/person-years	87/51373	66/50708	77/50630	75/49861	
Age, sex adjusted model ^a^	1 (Ref.)	0.89 (0.64–1.23)	1.06 (0.78–1.45)	1.05 (0.77–1.44)	0.561
Multiple adjusted model ^b^	1 (Ref.)	0.87 (0.63–1.21)	1.02 (0.74–1.39)	0.98 (0.71–1.35)	0.918
	**Water Use**				*p* for trend
	**Q1**	**Q2**	**Q3**	**Q4**	
N	4347	4347	4347	4346	
Deaths/person-years	91 /52107	62/50463	79/50567	73/49437	
Age, sex adjusted model ^a^	1 (Ref.)	0.80 (0.57–1.11)	0.89 (0.66–1.22)	0.87 (0.64–1.18)	0.488
Multiple adjusted model ^b^	1 (Ref.)	0.79 (0.57–1.09)	0.90 (0.66–1.23)	0.85 (0.62–1.16)	0.427
	**Land Use**				*p* for trend
	**Q1**	**Q2**	**Q3**	**Q4**	
N	4347	4347	4347	4346	
Deaths/person-years	76/49988	77/50326	73/51004	79/51254	
Age, sex adjusted model ^a^	1 (Ref.)	1.30 (0.94–1.80)	1.13 (0.81–1.57)	1.20 (0.86–1.66)	0.449
Multiple adjusted model ^b^	1 (Ref.)	1.22 (0.88–1.70)	1.06 (0.76–1.48)	1.10 (0.79–1.54)	0.789
	**Energy Use**				*p* for trend
	**Q1**	**Q2**	**Q3**	**Q4**	
N	4347	4347	4347	4346	
Deaths/person-years	91/52107	62/50463	79/50567	73/49437	
Age, sex adjusted model ^a^	1 (Ref.)	1.16 (0.84–1.60)	1.05 (0.76–1.47)	1.36 (0.99–1.86)	0.104
Multiple adjusted model ^b^	1 (Ref.)	1.14 (0.83–1.58)	0.97 (0.70–1.37)	1.30 (0.94–1.79)	0.221

^a^ Age was the underlying time variable. Adjusted for sex and stratified by age and year of entrance to the cohort. ^b^ Additionally adjusted for body mass index, adding a quadratic term, smoking, physical activity, time watching television, marital status, hypercholesterolemia and hypertension.

**Table 4:** Sensitivity analyses. Risk for all-cause mortality (hazard ratio and 95% confidence intervals) of the 17,387 participants of the SUN assessed in the current study, 1999–2019, according to quartiles of the energy-adjusted dietary greenhouse gas emissions.

		Energy-Adjusted Greenhouse Gas Emission				*p* for Trend
	Cases/Person-Years	Q1	Q2	Q3	Q4	
Overall	305/202,573	1 (Ref.)	0.87 (0.63–1.21)	1.02 (0.74–1.39)	0.98 (0.71–1.35)	0.918
Excluding participants with energy intake under percentile 1 or over percentile 99	336/223,402	1 (Ref.)	0.83 (0.61–1.12)	0.96 (0.71–1.30)	0.92 (0.68–1.25)	0.781
Excluding participants who have died in the first 4 years	239/202,431	1 (Ref.)	0.95 (0.66–1.38)	1.06 (0.74–1.51)	1.02 (0.71–1.48)	0.788
Censoring follow-up at 10 years	169/153,438	1 (Ref.)	0.90 (0.58–1.39)	1.10 (0.73–1.66)	0.75 (0.47–1.18)	0.396
Excluding death occurring ≤45 years	260/129,908	1 (Ref.)	0.92 (0.64–1.33)	1.07 (0.75–1.52)	1.14 (0.81–1.61)	0.355
Excluding deaths occurring >80 years	248/199,998	1 (Ref.)	0.81 (0.57–1.15)	0.79 (0.55–1.12)	0.88 (0.62–1.24)	0.430

Age was the underlying time variable. Adjusted for sex, body mass index, adding a quadratic term, smoking, physical activity, time watching television, marital status, hypercholesterolemia and hypertension, stratified by age and year of entrance to the cohort.

## Supplementary Materials

The following are available online at https://www.mdpi.com/2072-6643/12/8/2385/s1, Figure S1: flow chart for inclusion of the 17,387 participants of the SUN cohort assessed in the current study, 1999–2019. Table S1: main sources of information of the use of resources and greenhouse gas emissions of foods items collected in the food frequency questionnaire used in the SUN project. Table S2: food groupings (and serving sizes) of the foods collected in the food frequency questionnaire used in the SUN project. Figure S2: spline regression model of the relative risk of mortality (the dotted lines represent 95% confidence intervals) according to energy-adjusted dietary greenhouse gas of the 17,387 participants of the SUN project assessed in the current study, 1999–2019. Table S3: environmental impact, food groups’ consumption and nutrient intake of the 17,387 participants of the SUN cohort assessed in the current study, 1999–2019, according to energy-adjusted quartiles of dietary water use. Table S4: environmental impact, food groups’ consumption and nutrient intake of the 17,387 participants of the SUN cohort assessed in the current study, 1999–2019, according to energy-adjusted quartiles of dietary land use. Table S5: environmental impact, food groups’ consumption and nutrient intake of the 17,387 participants of the SUN cohort assessed in the current study, 1999–2019, according to energy-adjusted quartiles of dietary energy use. Table S6: stratified analyses. Risk for all-cause mortality (hazard ratio and 95% confident intervals (HR (95% CI))) of the highest versus the lowest quartile of the energy-adjusted dietary greenhouse gas emissions of the 17,387 participants of the SUN project assessed in the current study, 1999–2019. Table S7: Pearson correlation coefficients between energy-adjusted quartiles of the indicated environmental impact indicators.
