# Supplementary material for: Nutritional Quality and Health Effects of Low Environmental Impact Diets: The “Seguimiento Universidad de Navarra” (SUN) Cohort
Source: Nutrients. 2020 Aug 9;12(8):2385. doi: 10.3390/nu12082385 (PMC7468973; doi:10.3390/nu12082385)
Supplement: Supplementary file 1 [file nutrients-12-02385-s001.pdf]

Supplementary Figure 1. Flow chart for inclusion of the 17,387 participants of the SUN cohort assessed in the current study, 1999-2019.

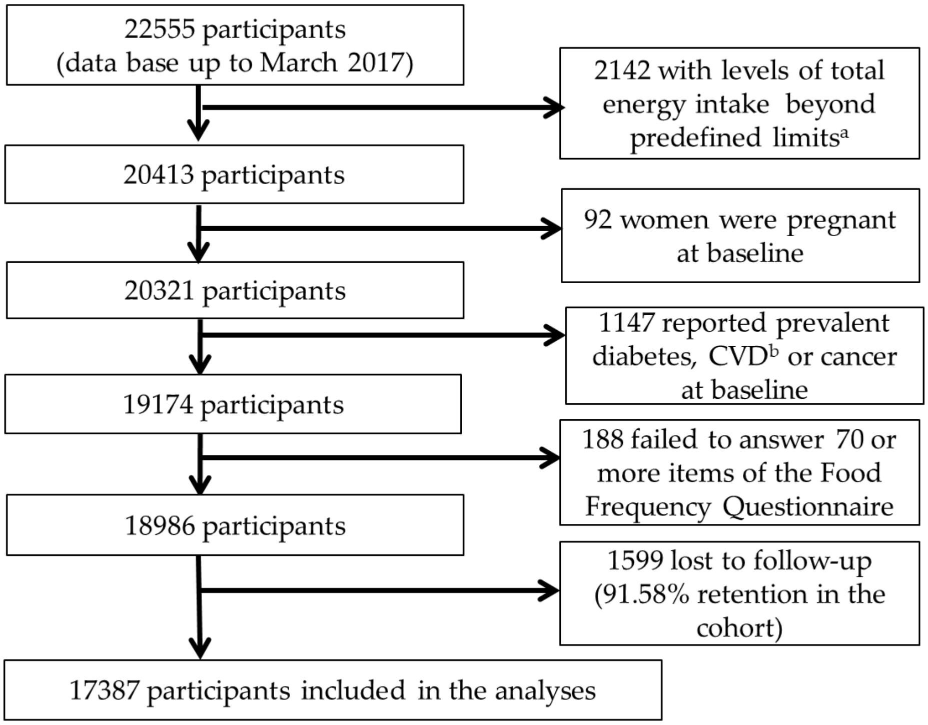

[a] <800 Kcal/day and <500 Kcal/day or >4000 Kcal/day and >3500 Kcal/day in men and women, respectively

[b]CVD: cardiovascular disease

Supplementary Table 1: Main sources of information of the use of resources and greenhouse gas emissions of foods items collected in the food frequency questionnaire used in the SUN project.

GREENHOUSE GAS EMISSION:

- CleanMetrics Corporation. Food Carbon emission calculator (2011) Available: http://www.foodemissions.com/foodemissions/Calculator.aspx [accessed September 2016].

- Nielsen PH, Nielsen AM, Weidema BP *et al.* (2003). LCA food data base. Available: www.lcafood.dk [accessed September 2016].

WATER USE:

- Herldbo J (2014) Recirculated aquaculture systems. Advantages and disadvantages. Good Practice Workshop, Copenhagen, Denmark. Available: https://circabc.europa.eu/sd/a/6112e063-d8aa-4533-9fbb-2abd47cce769/Presentation%204%20Jesper%20Heldbo%20EU_Baltic_Recirculated%20Aquaculture_JH.pdf [accessed September 2016].

- Hoekstra AY (2008) Water footprint of food. Available: http://waterfootprint.org/media/downloads/Hoekstra-2008-WaterfootprintFood.pdf [accessed September 2016].
- Hoekstra AY (2012) The hidden water resource use behind meat and dairy. *Animal frontiers* **2**, 3 - 8. ISSN 2160-6056. Available: http://purl.utwente.nl/publications/81617 [accessed September 2016].

- Mekonnen MM & Hoekstra AY (2011) The green, blue and grey water footprint of crops and derived crop products. Hydrology and Earth System Sciences. *Hydrol Earth Syst Sci* **15**,1577–1600.

- Ministerio de Medio Ambiente y Medio Rural y Marino: (Ministry of environment, rural and marine, Spain) (2006) Guía de Mejores Técnicas Disponibles en España del sector de Productos del mar (Best Available Techniques Guide of Sea products sector in Spain). Spain. Available: http://www.prtres.es/data/images/Gu%C3%ADa%20MTD%20en%20Espa%C3%B1a%20del%20sector%20de%20productos%20del%20mar-3D0CDD9B58C62B31.pdf [accessed September 2016].

LAND USE:

- Food and Agriculture Organization of the United Nations: data base 2009 FAOSTAT Available: www.faostat.fao.org/ [accessed September 2016].

- Cederberg C & Mattsson B (2000) Lifecycle assessment of milk production – a comparison of conventional and organic farming. *Journal of Cleaner Production* **8**, 49-60.

- Nielsen PH, Nielsen AM, Weidema BP *et al.* (2003) LCA food data base. Available: www.lcafood.dk [accessed September 2016].

ENERGY USE:

- Carlsson-Kanyama A & Faist M. Energy use in the food sector: a data survey Available: http://citeseerx.ist.psu.edu/viewdoc/download?rep=rep1&type=pdf&doi=10.1.1.205.8375 [accessed September 2016].

- Foster C, Green K, Bleda M *et al.* (2006) Environmental Impacts of food production and consumption: A report to the Department for environment, food and rural affair. Manchester Business School. Defra, London. Available: http://www.ifr.ac.uk/waste/Reports/DEFRA-Environmental%20Impacts%20of%20Food%20Production%20%20Consumption.pdf [accessed September 2016].
- Garrido A, Bardají I, De Blas C *et al.* (2011) Indicadores de sostenibilidad de la agricultura y ganadería españolas (Spanish agriculture and livestock indicators of sustainability). Available: http://www.eurocarne.com/daal?a1=informes&a2=Informe_final_7868.pdf [accessed September 2016].

- Gołaszewski J, de Visser CLM, Brodziński Z *et al.* (2012) State of the Art on Energy Efficiency in Agriculture. Country data on energy consumption in different agroproduction sectors in the European countries. Available: http://www.agree.aua.gr/files/agree_state.pdf [accessed September 2016].

- Hambly, J (2011) Environmental – Ecological Impact of the Dairy Sector (Literature Review on Dairy Products for an Inventory of Key Issues – List of Environmental Initiatives and Influences on the Dairy Sector*). International Journal of Dairy Technology* **64**, 145–146.

- Hornborg S, Ziegler F (2014) Aquaculture and energy use: a desk-top study. Available: http://vbcv.science.gu.se/digitalAssets/1536/1536133_publication---energy-use-in-aquaculture.pdf [accessed September 2016].

- Masanet E, Therkelsen P, Worrell E (2012) Energy Efficiency Improvement and Cost Saving Opportunities for the Baking Industry. An ENERGY STAR® Guide for Plant and Energy Managers. Available: https://www.energystar.gov/sites/default/files/buildings/tools/Baking_Guide.pdf [accessed September 2016].

Supplementary Table 2. Food groupings (and serving sizes) of the foods collected in the food frequency questionnaire used in the SUN project

Group	Food items
Dairy products	Whole milk (1 cup (200ml); Semi-skimmed milk (1 cup (200ml); Non-fat milk (1 cup (200ml); Sweetened condensed milk (1 spoon (15g)); Cream (½ cup (100g)); Milk shake (1 glass (200ml)); Yogurt (whole) (1 unit (125g)); Skimmed yogurt (1 unit (125g)); Petit Suisse Cheese (100g); Curd (½ cup (100g)); Cheese cream or cheese wedge (1 portion (30g)); Old cheese (50g); Fresh cheese (50g); Custard (1 cup (200ml)); Ice cream (1 unit (100g))
Eggs	Eggs (1 unit (60g))
All types of meat	Processed meat; Red meat; White meat
Processed meat	Cured ham (50g); Boiled ham (50g); Meat products (Mortadella salami, bologna, cured meats and cold cuts) (50 g); Sausages (50g); Foie-gras, Pâté (25g); Blood sausage (50g); Meatballs, soft pork sausage (50g); Bacon (50g)
Red meat	Beef or veal meat (150g); Pork meat (150g); Lamb meat (150g); Liver (100g); Entrails (100g)
White meat	Chicken or turkey with/without skin (150g); Rabbit (150g)
Fish and seafood	White fish (150g); Blue fish (150g); Cod (150g); Salad or smoked fish (50g); Clam, oyster, mussels (60g); Prawn, king prawn, crayfish (100g); Octopus, squid, cuttlefish (150g)
Vegetables	Spinach, Swiss chard (250g); Cabbage , cauliflower, Brussels sprouts (250g); Lettuce, endive (250g); Tomato (1 piece (150g)); Carrot, pumpkin (250g); Green bean (250g); Eggplant, zucchini, cucumber (250g); Peppers; Asparagus (250g); Gazpacho (250g); Other vegetables (250g); French fries (1portion (150g)); Roast or boiled potatoes (150g)
Fresh fruit	Oranges, grapefruit, tangerine (1 orange, 1 grapefruit, 2 tangerines (200g)); Banana (1 piece (150g)); Apple, pear (1 piece (175g)); Strawberry (6 units, dessert plate (60g)) ; Peach, apricot, nectarine (1 piece (150g)); Cherry, plum (1 dessert plate (150g)); Fig, early fig/black fig (2 portions (150g)); Watermelon (200-250g) ; Melon (200-250g); Grapes (1 bunch, dessert plate (150g)); Mango, papaya (1piece (150g)); Kiwi (1piece (150g))
Processed fruit	Fruit in syrup (2 units (150g)); Fruit in its own juice (2 units (150g)); Date, dry fig, prune, current (150g)
Nuts	Almonds, peanuts, hazelnuts, walnuts (50g)
Legumes	Lentils (60g dry); Chickpeas (60g dry); Beans (60g dry); Peas (60g dry)
Cereals	White bread (3 slices (60g)); Whole grain bread (3 slices (60g)); Cold breakfast cereal (30g); Rice (60g dry); Pasta (60g dry); Pizza (1 portion (200g))
Pastry products	Simple cookies (4-6 units (50g)); Cookies with chocolate filling (4-6 units (50g)); Muffins (1-2 units (45g)); Donuts (1 unit (50 g)); Industrial bakery, croissants (1 unit (50 g)); Home made pastries (50g); Cakes (50g); Cruller (100g); Chocolates (30g); Marzipan, bun, tea cookies (1 portion (90g)

Group	Food items
Oils and fats	Olive oil (1 spoon (10g)); Sunflower oil (1 spoon (10g)); Corn oil (1 spoon (10g)); Other oils (1 spoon (10g)); Margarine (Individual portion (10g)); Butter (Individual portion (10g)); Lard (Individual portion (10g))

Supplementary Figure 2. Spline regression model of the relative risk of mortality (the dotted lines represent 95% confidence intervals) according to energy-adjusted dietary greenhouse gas of the 17,387 participants of the SUN project assessed in the current study, 1999-2019.

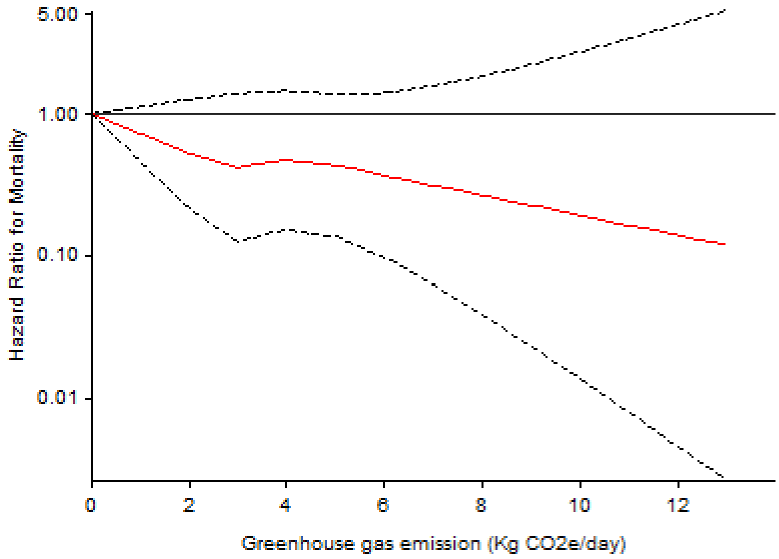

CO_2e: carbon dioxide equivalents

Age was the underlying time variable. Adjusted for sex, body mass index (BMI), quadratic term for BMI, smoking, physical activity, length of television watching, marital status, hypercholesterolemia and hypertension, and stratified by age and year of entrance to the cohort.

Supplementary Table 3. Environmental impact, food groups´ consumption and nutrient intake of the 17,387 participants of the SUN cohort assessed in the current study, 1999-2019, according to energy-adjusted quartiles of dietary water use.

	Energy-adjusted water use (L/day)					
	**Q1**<br>**1464-3372**	**Q2**<br>**3373-3726**	**Q3**<br>**3727-4110**	**Q4**<br>**4111-10548**	**p trend***	**p value ‡**
Frequency (n)	4,347	4,347	4,347	4,346		
Environmental impact data						
GHG[a] emission (kg CO_2e[b]/day), mean (SD)	2.90 (0.88)	3.19 (0.88)	3.68 (0.89)	4.40 (1.07)	<0.001	<0.001
Water use (L/day), mean (SD)	3147 (813)	3421 (803)	3889 (794)	4558 (929)	<0.001	<0.001
Land use (m^2/day), mean (SD)	6.74 (1.98)	6.69 (1.86)	7.28 (1.86)	7.97 (1.89)	<0.001	<0.001
Energy use (Megajoules/day), mean (SD)	16.4 (4.6)	16.6 (4.3)	17.8 (4.2)	19.6 (4.7)	<0.001	<0.001
**Food (servings/day)[c]**						
Dairy products	2.72 (1.53)	2.83 (1.50)	3.05 (1.65)	3.38 (1.95)	<0.001	<0.001
Eggs	2.66 (0.96)	2.70 (0.88)	2.79 (0.87)	2.82 (0.92)	<0.001	<0.001
All types of meats	1.41 (0.73)	1.68 (0.72)	1.95 (0.76)	2.38 (0.94)	<0.001	<0.001
Processed meat	0.32 (0.21)	0.43 (0.23)	0.58 (0.26)	0.77 (0.33)	<0.001	<0.001
Red meat	0.23 (0.18)	0.29 (0.20)	0.32 (0.21)	0.39 (0.30)	<0.001	<0.001
White meat	0.87 (0.59)	0.96 (0.59)	1.05 (0.63)	1.21 (0.76)	<0.001	<0.001
Fish and seafood	0.69 (0.50)	0.69 (0.41)	0.72 (0.38)	0.75 (0.42)	<0.001	<0.001
Vegetables	2.47 (1.34)	2.55 (1.35)	2.67 (1.46)	2.96 (1.81)	<0.001	<0.001
Fresh fruit	2.00 (1.60)	2.21 (1.61)	2.44 (1.87)	2.85 (2.49)	<0.001	<0.001
Processed fruit	0.10 (0.23)	0.11 (0.20)	0.11 (0.22)	0.11 (0.27)	0.453	0.846
Nuts	0.13 (0.20)	0.14 (0.22)	0.15 (0.24)	0.16 (0.28)	<0.001	<0.001
Legumes	0.40 (0.37)	0.37 (0.28)	0.37 (0.27)	0.37 (0.26)	<0.001	<0.001
Cereals	2.36 (1.56)	1.86 (1.19)	1.78 (1.15)	1.55 (1.02)	<0.001	<0.001
Pastry products	1.28 (1.16)	0.96 (0.81)	0.95 (0.79)	0.85 (0.76)	<0.001	<0.001
Oils and fats	1.85 (1.53)	1.84 (1.49)	1.9 (1.52)	2.00 (1.61)	<0.001	<0.001
**Macronutrient intake**						
Carbohydrates (% of energy)	47 (7)	44 (7)	42 (7)	40 (7)	<0.001	<0.001
Protein (% of energy)	16 (3)	18 (3)	19 (3)	20 (3)	<0.001	<0.001

Fat (% of energy)	35 (6)	36 (6)	37 (6)	38 (7)	<0.001	<0.001
Saturated fatty acids (% of energy)	12 (3)	12 (3)	13 (3)	13 (3)	<0.001	<0.001
Cholesterol (mg/d)	383 (136)	390 (131)	426 (143)	464 (167)	<0.001	<0.001
Monounsaturated fatty acids (% of energy)	15 (4)	16 (4)	16 (4)	17 (8)	<0.001	<0.001
Polyunsaturated fatty acids (% of energy)	5.5 (1.8)	5.2 (1.5)	5.0 (1.4)	4.9 (1.3)	<0.001	<0.001
Omega 3 (mg/d)	2.71 (1.53)	2.53 (1.15)	2.60 (1.09)	2.65 (1.09)	0.161	<0.001
Dietary fibre intake (g/day)	27.6 (11.9)	26.8 (11)	27.7 (11.7)	29.5 (13.6)	<0.001	<0.001
Added sugars (g/day)	61.8 (32.9)	53.9 (28.2)	54.5 (28.7)	52.0 (29.0)	<0.001	<0.001
**Micronutrient intake**						
Vitamins						
Vitamin A (μg/d)	1820 (1309)	1903 (1312)	1979 (1373)	2180 (1677)	<0.001	<0.001
Folic acid (μg/d)	371 (159)	389 (152)	413 (166)	462 (199)	<0.001	<0.001
Vitamin B12 (μg/d)	8.43 (4.81)	8.96 (4.31)	9.72 (4.66)	10.84 (5.44)	<0.001	<0.001
Vitamin C (mg/d)	245 (130)	268 (134)	285 (153)	323 (179)	<0.001	<0.001
Vitamin D (μg/d)	6.31 (5.21)	6.03 (4.11)	6.06 (3.93)	6.18 (4.26)	0.251	0.011
Vitamin E (mg/d)	7.17 (3.93)	6.76 (3.31)	6.87 (3.37)	7.01 (3.50)	0.135	<0.001
Minerals						
Iron (mg/d)	16.4 (4.9)	16.2 (4.6)	17.1 (4.8)	18.5 (5.3)	<0.001	<0.001
Zinc (mg/d)	14.7 (7.5)	16.4 (9.4)	18.3 (10.5)	21.9 (13.8)	<0.001	<0.001
Potassium (mg/d)	4429 (1432)	4528 (1400)	4812 (1468)	5318 (1772)	<0.001	<0.001
Sodium (mg/d)	3564 (2722)	3216 (2107)	3247 (1957)	3276 (1934)	<0.001	<0.001
Calcium (mg/d)	1138 (427)	1164 (412)	1240 (458)	1355 (528)	<0.001	<0.001

*Linear trends were analyzed by introducing the quartiles of the index as continuous variables. $p<0.05$ was considered statistically significant

ł Analysis of variance (ANOVA) tests were utilized to compare quartiles. $p<0.05$ was considered statistically significant

[a] GHG: greenhouse gas

[b] CO_2e: carbon dioxide equivalents

[c]Specific food products, and their serving sizes, collected in each food group is described in Supplementary table 2.

Supplementary Table 4. Environmental impact, food groups´ consumption and nutrient intake of the 17,387 participants of the SUN cohort assessed in the current study, 1999-2019, according to energy-adjusted quartiles of dietary land use.

	Energy-adjusted land use (m^2/day)					
	**Q1**<br>**2.80-6.49**	**Q2**<br>**6.50-7.09**	**Q3**<br>**7.10-7.73**	**Q4**<br>**7.74-20.68**	**p trend***	**p value ∤**
Frequency (n)	4,347	4,347	4,347	4,346		
Environmental impact data						
GHG[a] emission (kg CO_2e[b]/day), mean (SD)	3.13 (0.98)	3.34 (0.97)	3.62 (1.01)	4.07 (1.17)	<0.001	<0.001
Water use (L/day), mean (SD)	3508 (937)	3541 (942)	3769 (932)	4197 (1005)	<0.001	0.220
Land use (m^2/day), mean (SD)	6.24 (1.61)	6.58 (1.65)	7.20 (1.65)	8.65 (2.00)	<0.001	<0.001
Energy use (Megajoules/day), mean (SD)	16.9 (4.6)	16.8 (4.4)	17.5 (4.3)	19.2 (4.7)	<0.001	<0.001
**Food (servings/day)[c]**						
Dairy products	2.62 (1.48)	2.79 (1.44)	3.02 (1.59)	3.55 (2.02)	<0.001	<0.001
Eggs	2.73 (0.96)	2.76 (0.89)	2.76 (0.88)	2.72 (0.92)	0.461	<0.001
All types of meats	1.64 (0.87)	1.78 (0.81)	1.93 (0.86)	2.07 (0.89)	<0.001	<0.001
Processed meat	0.39 (0.26)	0.49 (0.28)	0.56 (0.30)	0.65 (0.34)	<0.001	<0.001
Red meat	0.30 (0.22)	0.31 (0.22)	0.31 (0.24)	0.32 (0.25)	<0.001	<0.001
White meat	0.95 (0.68)	0.98 (0.61)	1.06 (0.66)	1.10 (0.67)	<0.001	<0.001
Fish and seafood	0.77 (0.51)	0.72 (0.42)	0.69 (0.38)	0.69 (0.39)	<0.001	<0.001
Vegetables	2.88 (1.64)	2.62 (1.42)	2.57 (1.41)	2.59 (1.57)	<0.001	<0.001
Fresh fruit	2.87 (2.34)	2.32 (1.84)	2.20 (1.69)	2.11 (1.78)	<0.001	<0.001
Processed fruit	0.16 (0.31)	0.10 (0.23)	0.09 (0.17)	0.08 (0.18)	<0.001	<0.001
Nuts	0.21 (0.33)	0.14 (0.21)	0.12 (0.18)	0.12 (0.18)	<0.001	<0.001
Legumes	0.38 (0.29)	0.38 (0.27)	0.37 (0.28)	0.38 (0.34)	0.633	0.290
Cereals	2.38 (1.53)	1.90 (1.21)	1.70 (1.11)	1.58 (1.07)	<0.001	<0.001
Pastry products	0.85 (0.83)	0.88 (0.78)	0.97 (0.83)	1.33 (1.08)	<0.001	<0.001
Oils and fats	2.14 (1.66)	1.80 (1.42)	1.79 (1.43)	1.86 (1.60)	<0.001	<0.001
**Macronutrient intake**						
Carbohydrates (% of energy)	47 (7)	44 (7)	42 (7)	41 (7)	<0.001	<0.001
Protein (% of energy)	17 (3)	18 (3)	19 (3)	19 (3)	<0.001	<0.001

Fat (% of energy)	35 (7)	36 (6)	37 (6)	38 (6)	<0.001	<0.001
Saturated fatty acids (% of energy)	11 (3)	12 (3)	13 (3)	14 (3)	<0.001	<0.001
Cholesterol (mg/d)	387 (147)	401 (137)	420 (144)	454 (157)	<0.001	<0.001
Monounsaturated fatty acids (% of energy)	15 (4)	16 (4)	16 (3)	16 (3)	<0.001	<0.001
Polyunsaturated fatty acids (% of energy)	5.3 (1.7)	5.2 (1.5)	5.2 (1.5)	5.0 (1.4)	<0.001	<0.001
Omega 3 (mg/d)	2.76 (1.42)	2.53 (1.16)	2.53 (1.16)	2.67 (1.15)	0.007	<0.001
Dietary fibre intake (g/day)	32.1 (13.9)	27.2 (11.1)	26.2 (10.9)	26 (11.3)	<0.001	<0.001
Added sugars (g/day)	57.4 (32.3)	53.2 (28.1)	53.4 (27.9)	58.1 (31.1)	0.099	<0.001
**Micronutrient intake**						
Vitamins						
Vitamin A (µg/d)	2221 (1614)	1929 (1377)	1856 (1288)	1875 (1399)	<0.001	<0.001
Folic acid (µg/d)	441 (185)	398 (163)	394 (167)	403 (174)	<0.001	<0.001
Vitamin B12 (µg/d)	9.30 (5.20)	9.33 (4.79)	9.39 (4.51)	9.93 (5.07)	<0.001	<0.001
Vitamin C (mg/d)	311 (174)	273 (140)	268 (140)	268 (152)	<0.001	<0.001
Vitamin D (µg/d)	6.97 (5.5)	6.06 (4.01)	5.80 (3.86)	5.75 (3.93)	<0.001	<0.001
Vitamin E (mg/d)	7.76 (4.19)	6.64 (3.15)	6.64 (3.35)	6.77 (3.23)	<0.001	<0.001
Minerals						
Iron (mg/d)	18.0 (5.4)	16.6 (4.8)	16.5 (4.8)	17.0 (4.9)	<0.001	<0.001
Zinc (mg/d)	16.8 (9.6)	16.9 (10.0)	17.7 (10.3)	19.9 (13.0)	<0.001	<0.001
Potassium (mg/d)	5035 (1689)	4646 (1500)	4625 (1471)	4780 (1552)	<0.001	<0.001
Sodium (mg/d)	3362 (2628)	3183 (1984)	3345 (2100)	3412 (2054)	0.038	<0.001
Calcium (mg/d)	1140 (394)	1148 (399)	1216 (433)	1394 (570)	<0.001	<0.001

*Linear trends were analyzed by introducing the quartiles of the index as continuous variables. $p<0.05$ was considered statistically significant.

ɫ Analysis of variance (ANOVA) tests were utilized to compare quartiles. $p<0.05$ was considered statistically significant.

[a] GHG: greenhouse gas

[b] CO_2e: carbon dioxide equivalents

[c]Specific food products, and their serving sizes, collected in each food group is described in Supplementary table 2.

Supplementary Table 5. Environmental impact, food groups´ consumption and nutrient intake of the 17,387 participants of the SUN cohort assessed in the current study, 1999-2019, according to energy-adjusted quartiles of dietary energy use

	**Energy-adjusted energy use (Megajoules/day)**					
	**Q1**<br>**0.25-15.77**	**Q2**<br>**15.78-17.42**	**Q3**<br>**17.43-19.19**	**Q4**<br>**19.20- 41.46**	**p trend***	**p value ⱡ**
Frequency (n)	4,347	4,347	4,347	4,346		
Environmental impact data						
GHG[a] emission (kg CO_2e[b]/day), mean (SD)	2.96 (0.88)	3.30 (0.88)	3.63 (0.94)	4.28 (1.18)	<0.001	<0.001
Water use (L/day), mean (SD)	3545 (976)	3568 (930)	3753 (913)	4150 (1028)	<0.001	<0.001
Land use (m^2/day), mean (SD)	6.95 (2.01)	6.86 (1.88)	7.12 (1.86)	7.74 (1.99)	<0.001	<0.001
Energy use (Megajoules/day), mean (SD)	14.7 (3.6)	16.2 (3.6)	17.9 (3.5)	21.6 (4.5)	<0.001	<0.001
**Food (servings/day)[c]**						
Dairy products	2.37 (1.27)	2.71 (1.40)	3.03 (1.47)	3.87 (2.09)	<0.001	<0.001
Eggs	2.59 (0.93)	2.73 (0.88)	2.79 (0.88)	2.86 (0.93)	<0.001	<0.001
All types of meats	1.51 (0.76)	1.75 (0.74)	1.95 (0.82)	2.20 (0.99)	<0.001	<0.001
Processed meat	0.41 (0.26)	0.50 (0.28)	0.55 (0.30)	0.62 (0.36)	<0.001	<0.001
Red meat	0.25 (0.18)	0.28 (0.19)	0.33 (0.22)	0.38 (0.30)	<0.001	<0.001
White meat	0.85 (0.59)	0.97 (0.58)	1.07 (0.64)	1.20 (0.77)	<0.001	<0.001
Fish and seafood	0.53 (0.30)	0.63 (0.34)	0.73 (0.35)	0.97 (0.55)	<0.001	<0.001
Vegetables	2.80 (1.68)	2.53 (1.37)	2.57 (1.42)	2.75 (1.56)	0.406	<0.001
Fresh fruit	2.82 (2.55)	2.27 (1.77)	2.20 (1.64)	2.21 (1.62)	<0.001	<0.001
Processed fruit	0.11 (0.23)	0.10 (0.19)	0.10 (0.21)	0.11 (0.29)	0.208	0.001
Nuts	0.21 (0.33)	0.14 (0.21)	0.13 (0.19)	0.11 (0.17)	<0.001	<0.001
Legumes	0.42 (0.40)	0.37 (0.25)	0.36 (0.24)	0.36 (0.27)	<0.001	<0.001
Cereals	2.33 (1.52)	1.84 (1.14)	1.71 (1.05)	1.68 (1.26)	<0.001	<0.001
Pastry products	1.15 (1.06)	1.04 (0.91)	0.98 (0.84)	0.87 (0.78)	<0.001	<0.001
Oils and fats	2.49 (1.85)	1.87 (1.47)	1.67 (1.32)	1.57 (1.27)	<0.001	<0.001
**Macronutrient intake**						
Carbohydrates (% of energy)	46 (7)	44 (7)	42 (6)	41 (8)	<0.001	<0.001

Protein (% of energy)	16 (2)	18 (2)	19 (3)	21 (3)	<0.001	<0.001
Fat (% of energy)	36 (7)	37 (6)	37 (6)	37 (6)	<0.001	<0.001
Saturated fatty acids (% of energy)	11 (3)	12 (3)	13 (3)	13 (4)	<0.001	<0.001
Cholesterol (mg/d)	354 (122)	395 (127)	427 (134)	487 (172)	<0.001	<0.001
Monounsaturated fatty acids (% of energy)	16 (4)	16 (4)	16 (3)	15 (3)	<0.001	<0.001
Polyunsaturated fatty acids (% of energy)	5.5 (1.8)	5.3 (1.5)	5.1 (1.4)	4.9 (1.3)	<0.001	<0.001
Omega 3 (mg/d)	2.40 (1.23)	2.48 (1.20)	2.60 (1.07)	3.01 (1.31)	<0.001	<0.001
Dietary fibre intake (g/day)	31.6 (14.1)	26.8 (10.9)	26.2 (11.0)	27.0 (11.5)	<0.001	<0.001
Added sugars (g/day)	53.7 (29.2)	54.2 (28.6)	54.8 (28.6)	59.5 (33.1)	<0.001	<0.001
**Micronutrient intake**						
Vitamins						
Vitamin A (µg/d)	2103 (1626)	1843 (1243)	1863 (1331)	2072 (1479)	0.689	<0.001
Folic acid (µg/d)	412 (188)	384 (154)	398 (164)	440 (181)	<0.001	<0.001
Vitamin B12 (µg/d)	7.20 (3.44)	8.59 (3.85)	9.78 (4.26)	12.37 (6.09)	<0.001	<0.001
Vitamin C (mg/d)	292 (168)	265 (142)	272 (147)	291 (152)	0.673	<0.001
Vitamin D (µg/d)	4.48 (2.99)	5.32 (3.32)	6.23 (3.82)	8.56 (5.82)	<0.001	<0.001
Vitamin E (mg/d)	7.92 (4.47)	6.81 (3.39)	6.48 (3.02)	6.59 (2.88)	<0.001	<0.001
Minerals						
Iron (mg/d)	17.2 (5.3)	16.2 (4.6)	16.7 (4.7)	18.1 (5.2)	<0.001	<0.001
Zinc (mg/d)	15 (7.4)	15.8 (8.2)	17.6 (9.3)	23 (15.1)	<0.001	<0.001
Potassium (mg/d)	4816 (1739)	4528 (1447)	4668 (1442)	5074 (1557)	<0.001	<0.001
Sodium (mg/d)	3135 (1973)	3168 (2091)	3316 (2015)	3684 (2643)	<0.001	<0.001
Calcium (mg/d)	1092 (381)	1135 (397)	1221 (419)	1450 (561)	<0.001	<0.001

*Linear trends were analyzed by introducing the quartiles of the index as continuous variables. p<0.05 was considered statistically significant

ł Analysis of variance (ANOVA) tests were utilized to compare quartiles. p<0.05 was considered statistically significant

[a] CO_2e: carbon dioxide equivalents

[b] Specific food products, and their serving sizes, collected in each food group is described in Supplementary table

Supplementary Table 6. Stratified analyses. Risk for all-cause mortality (Hazard Ratio and 95% confident intervals (HR (95% CI))) of the highest versus the lowest quartile of the energy-adjusted dietary greenhouse gas emissions of the 17,387 participants of the SUN project assessed in the current study, 1999-2019.

	**N**	**Deaths (N)**	**HR (95% CI)**	***p* for trend***	***p* for interaction***
Overall	17,387	305	0.98 (0.71-1.35)	0.918	
Including only participants aged >45 years	4,417	226	1.11 (0.77-1.60)	0.299	0.099
Including only participants aged ≤45 years	12,970	79	0.60 (0.30-1.22)	0.170	
Including only men	10,556	94	1.04 (0.55-1.97)	0.888	0.849
Including only women	6,831	211	0.93 (0.63-1.36)	0.995	
Including only more active[a,b] participants	8,693	135	1.18 (0.72-1.94)	0.489	0.925
Including only more sendentary[a,c] participants	8,694	170	0.90 (0.58-1.40)	0.885	
Including only participants with BMI<25 kg/m^2	12,284	143	1.08 (0.66-1.79)	0.743	0.472
Including only participants with BMI≥25 kg/m^2	5,103	162	0.83 (0.54-1.28)	0.743	

Age was the underlying time variable.

Adjusted for sex, body mass index (BMI), quadratic term for BMI, smoking, physical activity, length of television watching, marital status, hypercholesterolemia and hypertension, and stratified by age and year of entrance to the cohort.

*p<0.05 were considered statistically significant

[a]Physical activity median=2.27 Mets-h/day

[b]Physical activity>median

[c]Physical activity≤median

Supplementary Table 7. Pearson correlation coefficients between energy-adjusted quartiles of the indicated environmental impact indicators.

	**Greenhouse gas emission**	**Water use**	**Land use**	**Energy use**
**Greenhouse gas emission**	1.00			
**Water use**	0.81	1.00		
**Land use**	0.70	0.80	1.00	
**Energy use**	0.77	0.75	0.74	1.00
